# Effect of Mono- and Polysaccharide on the Structure and Property of Soy Protein Isolate during Maillard Reaction

**DOI:** 10.3390/foods13172832

**Published:** 2024-09-06

**Authors:** Kun Wen, Qiyun Zhang, Jing Xie, Bin Xue, Xiaohui Li, Xiaojun Bian, Tao Sun

**Affiliations:** College of Food Science & Technology, Shanghai Ocean University, Shanghai 201306, China; wenkun_wen@163.com (K.W.); 19821253356@163.com (Q.Z.); jxie@shou.edu.cn (J.X.); bxue@shou.edu.cn (B.X.); xhli@shou.edu.cn (X.L.); xjbian@shou.edu.cn (X.B.)

**Keywords:** Maillard reaction, soy protein isolate, D-galactose, oat β-glucan, functional food

## Abstract

As a protein extracted from soybeans, soy protein isolate (SPI) may undergo the Maillard reaction (MR) with co-existing saccharides during the processing of soy-containing foods, potentially altering its structural and functional properties. This work aimed to investigate the effect of mono- and polysaccharides on the structure and functional properties of SPI during MR. The study found that compared to oat β-glucan, the reaction rate between SPI and D-galactose was faster, leading to a higher degree of glycosylation in the SPI–galactose conjugate. D-galactose and oat β-glucan showed different influences on the secondary structure of SPI and the microenvironment of its hydrophobic amino acids. These structural variations subsequently impact a variety of the properties of the SPI conjugates. The SPI–galactose conjugate exhibited superior solubility, surface hydrophobicity, and viscosity. Meanwhile, the SPI–galactose conjugate possessed better emulsifying stability, capability to produce foam, and stability of foam than the SPI–β-glucan conjugate. Interestingly, the SPI–β-glucan conjugate, despite its lower viscosity, showed stronger hypoglycemic activity, potentially due to the inherent activity of oat β-glucan. The SPI–galactose conjugate exhibited superior antioxidant properties due to its higher content of hydroxyl groups on its molecules. These results showed that the type of saccharides had significant influences on the SPI during MR.

## 1. Introduction

As a high-purity protein, soy protein isolate (SPI) contains nearly 20 types of amino acids, among which are essential amino acids. It is nutritious, cholesterol-free, and one of the few plant proteins that can replace animal proteins [1]. As the main component, soybean globulin accounts for more than 70% of soybean isolate proteins. These proteins possess specific amino acid sequences and three-dimensional structures, endowing them with particular functional properties [2]. Meanwhile, the spherical structure of SPI limits the exposure of hydrophobic residues, affecting its functional characteristics. This, in turn, restricts its application in the food industry [3].

Modifying SPI through various methods such as physical, enzymatic, and chemical treatments can significantly enhance its solubility, emulsifying properties, gelation, and nutritional value, thereby broadening its application in the food industry [4]. Among the various methods, the Maillard reaction (MR) has drawn much attention. The MR is a complicated non-enzymatic process of browning characterized by the interaction between the carbonyl groups of saccharides and the amino groups of proteins or amino acids. Given that MR occurs naturally without the requirement of additional chemicals, it is often seen as a perfect and hopeful method for enhancing protein characteristics [5,6]. The conditions of the MR, such as temperature, reaction time, and especially the type and structure of the co-existing saccharides, greatly influence the functional characteristics of SPI.

Previous studies have shown that monosaccharides can strengthen the functional characteristics of SPI. For example, D-glucose endowed SPI with a porous surface structure and increased hydrophilicity, consequently improving its water solubility and emulsifying property [7]. The MR resulted in the structural relaxation of SPI, with xylose and fructose enhancing its solubility. Notably, the SPI–fructose conjugate showed the highest solubility within 6 h of the reaction [8]. Maltose, being a classic disaccharide, contributes to the improved emulsion stability of SPI by increasing the steric hindrance among the droplets within the emulsion [9]. Compared with monosaccharides and oligosaccharides, the interaction of proteins with polysaccharides during the MR resulted in proteins with improved functional capabilities since polysaccharides possess stronger molecular steric hindrance [10]. Xanthan gum endowed SPI with enhanced foaming performance and the stability of the foam, and this improvement is associated with the proportion of SPI to xanthan gum in the MR process [11]. During the MR, both gum arabic (GA) and maltodextrin changed the secondary structure of SPI while exhibiting a strong capacity to enhance the emulsifying properties, with GA demonstrating a superior ability [12]. The conjugate of SPI with glucose and chitosan oligosaccharide displayed enhanced emulsifying properties when compared to the unmodified SPI. Furthermore, the SPI–glucose conjugate exhibited superior emulsifying properties [13]. Generally speaking, the SPI conjugates’ functional characteristics are correlated with the type and content of saccharides during the MR, and the conclusion was also confirmed by our previous work [14,15]. However, research on the impact of saccharides on the structure and function of SPI during the MR is still relatively limited.

D-galactose (Gal), as a six-carbon aldose, is one of the major monosaccharide components of soybean polysaccharides. During the MR, SPI interacted with Gal, thereby altering the protein’s structure and functional characteristics. Oat β-glucan (βG) is a polysaccharide composed of β-D-glucopyranose monomers linked by β-(1→3) and β-(1→4) glycosidic bonds. βG has demonstrated numerous physiological activities, with its hypoglycemic effect being the most notable [16]. Consequently, it is expected that βG could lead to SPI gaining hypoglycemic activity during the MR, thereby enhancing SPI’s functional properties. β-d-Glucopyranose is the structural unit of βG. Both D-galactose and β-d-glucopyranose are hexoses composed of six carbon atoms and an aldehyde group, and have the same D-configuration. It is curious how the structural link between them would differently affect the SPI’s structure and characteristics during the MR.

The aim of this work is to (1) reveal the influence of D-galactose and oat β-glucan on the structure of SPI during the MR, (2) evaluate the influence of D-galactose and oat β-glucan on SPI’s functionalities during the MR. Our findings offer new perspectives on using SPI in the food industry.

## 2. Materials and Methods

### 2.1. Materials

D-galactose, Fehling’s reagent, and soy protein isolate (purity: 90%) were sourced from Shanghai Maclin Biochemical Technology Co., Ltd. (Shanghai, China). A glucose assay kit was sourced from Shanghai Rongsheng Biopharmaceutical Co., Ltd. (Shanghai, China). α-Glucosidase (75.3 U/mg, from yeast) was sourced from Beijing Solaibao Biological Co., Ltd. (Beijing, China). 1,1-dipenyl-2-picrylhydrazyl (DPPH) and ferrozine were sourced from Sigma-Aldrich Co., Ltd. (Shanghai, China). The other reagents used for the work were of analytical grade.

### 2.2. Preparation of SPI-Gal and SPI-βG

A total of 8.0 g of Gal (βG) and 8.0 g of SPI were uniformly mixed using deionized water (200 mL). The mixed solution’s pH was regulated by NaOH (1 mol/L) up to 9.0. Then, the system was subjected to the reaction in condensation reflux (80 °C, 13 h). The pH, UV-Vis absorption (420 nm), and fluorescence value (emission wavelength: 420 nm, excitation wavelength: 347 nm) of the reaction system at 0, 1, 4, 7, 10, and 13 h were monitored. After 13 h, the reaction system was treated with ethanol to precipitate the product, which was washed repeatedly with ethanol until the filtrate did not contain Gal (βG). The products were freeze-dried to acquire the SPI conjugates: SPI-Gal (SPI-βG). A control group was established using SPI that had not undergone any treatment.

### 2.3. Characterization of SPI-Gal and SPI-βG

#### 2.3.1. Fourier Transform Infrared Spectroscopy (FTIR)

The FTIR spectra of the samples were determined by employing an FTIR spectrometer (Nicolet iS10, Thermos Scientific, USA) (scanning wavenumber: 500–4000 cm^−1^, resolution: 2 cm^−1^) [17]. The secondary structure was quantitatively estimated by PeakFit 4.12.

#### 2.3.2. Degree of Glycosylation (DG)

The DG was assayed by the previous method [18]. The O-phthalaldehyde (OPA) solution was obtained by mixing ethanol (3 mL, 95%) with OPA (120 mg), followed by the addition of Na_2_B_4_O_7_ (10 mmol/L, 75 mL, pH 9.7), SDS (20%, *w*/*w*, 7.5 mL), and β-mercaptoethanol (300 µL), and then adding deionized water to 150 mL. The sample (2 mg/mL) was dissolved in deionized water with a pH of 7.0. Then, the sample (300 µL) was mixed with OPA (6 mL) and incubated (5 min, 25 °C), and the absorbance (340 nm) was recorded. Deionized water was substituted for the sample solution as the blank. The DG was determined by the following equation:

(1) $$DG\left(\%\right)=\frac{D_{0}-D_{1}}{D_{0}}\times 100\%$$

where D_0_ and D_1_ represent the absorbance at 340 nm of the blank and sample, respectively.

#### 2.3.3. Fluorescence Spectra

The sample (0.1 mg/mL) was dissolved in phosphate-buffered solution (PBS) (0.01 mol/L, pH 7.0). A fluorescence spectrophotometer (FS-5, Edinburgh, UK) was used to record the fluorescence spectra (emission wavelength: 300–450 nm, excitation wavelength: 290 nm) of the samples [19].

### 2.4. Physicochemical Properties

#### 2.4.1. Determination of Surface Hydrophobicity (*H*_0_)

The *H*_0_ of the samples was assessed by a 1-anilino-8-naphthalenesulfonate (ANS) fluorescent probe [20]. ANS (8.0 mmol/L) was prepared using PBS (0.01 mol/L, pH 7.0). The samples (0.2, 0.4, 0.6, 0.8, and 1.0 mg/mL) were prepared using PBS (0.01 mol/L, pH 7.0). The fluorescence values (emission wavelength: 470 nm, excitation wavelength: 390 nm) were recorded by mixing the samples (3.0 mL) with ANS (30 μL). *H*_0_ was acquired by the slope of a fitted straight line.

#### 2.4.2. Solubility Measurement

The pH of the samples (2 mg/mL, 20 mL) was adjusted from 4 to 9 with HCl (0.5 mol/L) or NaOH (0.5 mol/L), and then centrifuged (12,000× *g*, 30 min). The supernatant and total protein content were assessed by Zheng’s method and the Kjeldahl method, respectively, and the ratio of the two was the solubility [15,21].

#### 2.4.3. Determination of Emulsifying Activity (EA) and Emulsifying Stability (ES)

The EA and ES were assayed by the previous reports with some modifications [22,23]. The sample (1 mg/mL) was prepared using deionized water with a pH of 7.0. The sample (12.0 mL) was mixed with soy oil (3.0 mL) and homogenized at 20,000 rpm for 2 min using a homogenizer (FJ200-SH, Huxi, China). The emulsion (50 μL) was taken from the bottom of the homogeneous emulsion at 0 (10) min. The emulsion was diluted with 5 mL of 0.1% (*w*/*v*) SDS solution, and then the absorbance (500 nm) was recorded. The EA and ES were acquired using the following equations:

(2) $$EA\left(m^{2}/g\right)=\frac{2\times 2.303\times A_{0}\times N}{c\times 1\times \emptyset \times 10,000}$$

(3) $$ES\left(min\right)=\frac{A_{0}}{A_{0}-A_{10}}\times 10$$

where 2 represents a coefficient used for unit conversion and calculation; 2.303 represents the approximate value of In 10; 1 represents the optical path (1 cm); 10,000 represents the unit conversion factor; 10 represents the time interval (10 min); c is the concentration of the sample (g/mL); ∅ represents the oil volume fraction (*v*/*v*) in the emulsion; A_0_ (A_10_) represents the absorbance at 0 (10) min; and N represents the dilution factor.

#### 2.4.4. Determination of Foaming Capacity (FC) and Foam Stability (FS)

The FC and FS were assayed by the previous method [24]. The SPI conjugates (1 g) were dissolved in phosphate-buffered solution (PBS) (pH 7.0, 0.2 mol/L, 30 mL) and homogenized (1 min, 20,000 rpm) using a homogenizer (FJ200-SH, Huxi, China). Subsequently, the volume (mL) of the foam was determined at 0 (30) min. The FC and FS were determined by the following equations:

(4) $$FC\left(\%\right)=\frac{V_{0}}{V_{a}}\times 100\%$$

(5) $$FS\left(\%\right)=\frac{V_{i}}{V_{0}}\times 100\%$$

where V_a_ and V_0_ (V_i_) represent the initial and homogenized 0 (30) min foam volume (mL), respectively.

### 2.5. Viscosity Measurement

The sample (80 mg/mL) was dissolved in deionized water with a pH of 7.0. The viscosity of the samples was evaluated using a rheometer (DHR-3, Waters Corporation, Milford, MA, USA) with cone and plate geometry (1.0 mm distance, 2°/60 mm) at 25 °C [25].

### 2.6. Physiological Properties

#### 2.6.1. Swelling Power (SP)

The samples (0.6 g) were diluted in deionized water (20 mL), stirred thoroughly (70 °C, 10 min), and boiled (15 min), then cooled and centrifuged (10 min, 642× *g*). The SP was expressed as the wet–dry ratio of the system [26].

#### 2.6.2. Fat-Binding Capacity (FB)

The SPI conjugates (400 mg) were added to soy oil (20 mL), stirred (60 min), and centrifuged (263× *g*, 30 min). The FB (g/g) is the ratio of the weight of the system after removing the supernatant to dry weight (400 mg) [25].

#### 2.6.3. Bile Acid-Binding Ability (BAB)

The BAB was measured according to previous reports with some modifications [25,27]. A bile acid solution was produced by mixing bile acid (300 mg) with NaOH (0.1 mol/L, 7.05 mL) and adding deionized water to 300 mL. The SPI conjugates (25 mg) were added to the bile acid solution (10 mL), stirred (2 h, 37 °C), and then strained using a filter (0.2 μm). The solution (1.0 mL), H_2_SO_4_ (16 mol/L, l5.0 mL), and furfuryl alcohol (0.9%, 1.0 mL) were combined and allowed to stand (5 min, 0 °C). The system was warmed (75 °C, 10 min), left to stand (3 min, 0 °C), and the absorbance (490 nm) was recorded. The control sample was prepared without the addition of the samples (SPI and its conjugates).

#### 2.6.4. Glucose Availability (GA) in Chemical Digestion

The sample (0.6 g) and glucose (0.6 g) were solubilized in deionized water (20 mL), mixed uniformly, and heated (70 °C, 10 min). After cooling, the system pH was regulated to 1.0–2.0 with HCl (0.1 mol/L). The system was incubated at 37 °C for 60 min to simulate gastric digestion. Then, the system pH was regulated to 6.8–7.2 with NaHCO_3_ (15 mg/mL) and incubated (30 min, 37 °C) to simulate duodenal digestion. After keeping the digestion simulation stationary for 20 min, the phase separation was obtained and then the supernatant was collected. The glucose content of the supernatant was evaluated using the glucose assay kit and the results were presented as mmol/L glucose [26].

#### 2.6.5. α-Glucosidase Inhibitory Activity

The α-glucosidase inhibitory activity was determined using a previous method with some modifications [28]. The sample solution (2–10 mg/mL) was prepared using PBS (0.2 mol/L, pH 6.8). The sample solution (0.2 mL), PBS (2 mL, 0.2 mol/L, pH 6.8), and α-glucosidase (0.2 mL, 0.2 U/mL) were mixed and incubated at 37 °C for 15 min. Then, p-nitrophenyl-α-D-glucopyranoside (pNPG) (2.5 mmol/L, 0.4 mL) was added to the reaction system and incubated at 37 °C for 15min. Finally, a Na_2_CO_3_ solution (0.8 mL, 0.2 mol/L) was added to the system to terminate the reaction. The absorbance of the system at 405 nm was measured. The α-glucosidase inhibition rate (%) was obtained using the following equation:

(6) $$\alpha -Glucosidase\;inhibition\;rate\left(\%\right)=\left(1-\frac{A_{i}-A_{j}}{A_{0}}\right)\times 100\%$$

where A_i_ represents the absorbance of the system after the reaction; A_0_ represents the absorbance of the buffer solution replacing the sample solution; and A_j_ represents the absorbance of the buffer solution replacing α-glucosidase.

### 2.7. Antioxidant Activities

#### 2.7.1. DPPH Scavenging Activity

The sample solution (0.5–3.5 mg/mL) was prepared using deionized water (pH 7.0). A DPPH ethanol solution (0.1 mmoL/L, 2.0 mL) was thoroughly mixed with the sample solution (2.0 mL), incubated under darkness (30 min), and the absorbance E_1_ was recorded (517 nm). Deionized water was substituted for the sample (E_0_) and ethanol was substituted for DPPH (E_2_) [29]. The DPPH scavenging rate (%) was determined using Formula (7), and a curve to fit the relationship between the concentration and DPPH scavenging rate was plotted. IC_50_ is defined as the concentration at which the DPPH scavenging rate reaches 50%.

(7) $$DPPH\;scavenging\;rate\left(\%\right)=\left(1-\frac{E_{1}-E_{2}}{E_{0}}\right)\times 100\%$$

#### 2.7.2. Determination of Reducing Power

The sample solution (3–10 mg/mL) was prepared using PBS (pH 6.60, 0.2 mol/L). The sample solution (2.5 mL), PBS (pH 6.60, 0.2 mol/L, 2.5 mL), and potassium ferricyanide (0.1 g/L, 2.5 mL) were thoroughly combined and incubated (50 °C, 20 min), and then combined with trichloroacetic acid (1 g/L, 2.5 mL) and centrifuged (411× *g*, 15 min). The supernatant (2.0 mL), FeCl_3_ (0.1%, *w*/*v*, 0.4 mL), and deionized water (2.0 mL) were combined. The absorbance of the system was measured at 700 nm against a blank (PBS (0.2 mol/L, pH 6.60) instead of the sample solutions) [29].

#### 2.7.3. Ferrous Ion (Fe^2+^)-Chelating Capacity

The sample solution (0.5–3.5 mg/mL) was prepared using deionized water (pH 7.0). The sample solution (1.0 mL), methanol (3.7 mL), and FeCl_2_ solution (0.1 mL, 2 mmol/L), were combined, and then ferrozine was added (0.2 mL, 5 mmol/L). The absorbance F_1_ (562 nm) was recorded after 10 min of standing. F_2_ is the absorbance of deionized water instead of ferrozine, and F_0_ is the absorbance of deionized water instead of the sample solution. The ferrous ion-chelating capacity (%) was determined using Formula (8), and a curve to fit the relationship between the concentration and ferrous ion-chelating capacity was plotted. EC_50_ is defined as the concentration at which the ferrous ion-chelating capacity reaches 50%.

(8) $${Fe}^{2+}\;chelating\;capacity\left(\%\right)=\left(1-\frac{F_{1}-F_{2}}{F_{0}}\right)\times 100\%$$

### 2.8. Statistical Analysis

Each experiment was conducted three times, and the value was reported as the average ± standard deviation. The data were subjected to one-way ANOVA by SPSS 27.0. Significance was determined when *p* < 0.05.

## 3. Results and Discussion

### 3.1. MR Process (pH, UV-Vis Absorbance, and Fluorescence Value)

**Figure 1:**
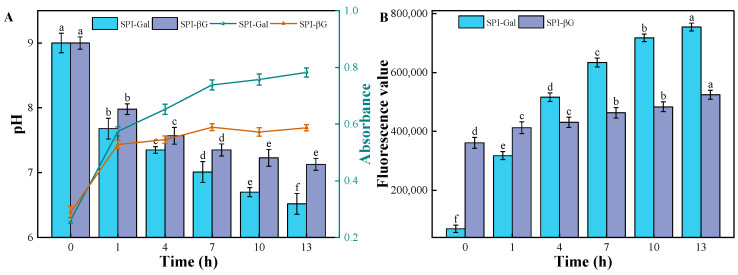
pH value, UV-Vis absorbance (**A**), and fluorescence value (**B**) of the Maillard reaction system. Different letters indicate statistically significant differences (*p* < 0.05).

The progress of the MR can be monitored by the pH value, UV-Vis absorbance, and fluorescence value [30]. With the increase in reaction time, the pH of the system gradually decreases (Figure 1). This is due to the generation of acidic compounds during the MR [31], while the UV-Vis absorbance value as well as the fluorescence value of the system gradually increased with the increase in heating time, further confirming the formation of soybean isolate protein couplings [32]. Additionally, the changes in the pH, UV-Vis absorbance, and fluorescence value of the SPI-Gal reaction system were more pronounced, indicating that the MR rate in this system was faster. It is evident that monosaccharides, such as galactose, are more reactive with SPI in the MR compared to polysaccharides like β-glucan. This could be due to several factors: for the same mass, galactose contains a greater number of carbonyl groups; and smaller molecules can more easily reach the amino groups of SPI with less steric hindrance. Li’s research suggested that monosaccharide–protein reaction systems exhibited a faster browning rate compared to those involving polysaccharides [33].

### 3.2. Structural Characterization

#### 3.2.1. Infrared Spectral Characterization and Secondary Structure

**Figure 2:**
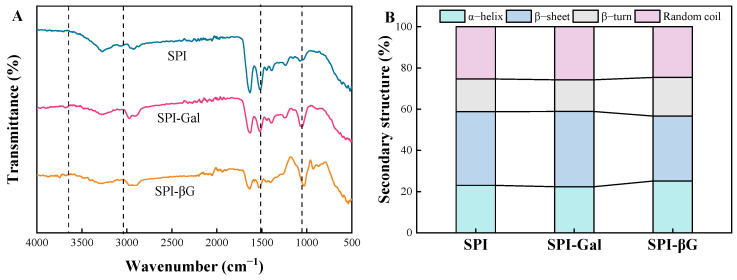
FT-IR spectra (**A**) and secondary structure (**B**) of SPI conjugates.

FT-IR spectroscopy is a common tool for investigating protein–carbohydrate conjugates [34]. As depicted in Figure 2A, the characteristic absorption peaks of SPI and its conjugates were located at 1520 cm^−1^, 1631 cm^−1^, and 1240 cm^−1^, representing amide II (N-H bending), amide I (C=O stretching), and amide III (C-N stretching and N-H deformation), respectively [35]. After MR, the conjugates exhibit broadened absorption peaks within the 3020–3682 cm^−1^ range, which was due to the introduction of hydroxyl groups from the sugar molecules that have covalently bonded to the SPI [36]. The absorption vibrations near 1036–1069 cm^−1^ were intensified in the conjugates compared to SPI, indicating an enhancement in the vibration of the C-N covalent bonds, which suggested that SPI is covalently linked with Gal/βG molecules [37]. During the MR, the reduction in the free amino groups of SPI led to a decrease in the absorption peak at 1523 cm^−1^, which was consistent with the report of Chu et al. [38].

Previous research showed that the amide I band is a broad absorption peak covering the range of 1600 to 1700 cm^−1^, which is actually a superposition of several sub-peaks representing α-helices, β-turns, random coils, and β-sheets [36,39,40]. As shown in Figure 2B, the content of α-helices and β-turns of SPI reduced during the MR with Gal. This phenomenon may be due to the MR affecting the stability of hydrogen bonds, which are the primary stabilizing forces within the α-helical structures of proteins [41]. Meanwhile, the content of the β-sheets and random coils of SPI-Gal increased, which might be due to the fact that the covalent binding of SPI with Gal enhanced the interaction between protein molecules [42]. After the MR with galactose, the conformation of SPI shifts from an ordered to a disordered state, thereby influencing its functional characteristics. In contrast, the α-helix and β-turn contents of the SPI-βG were raised, while the β-sheet and random coil contents decreased, indicating that the conformation of the conjugate shifted from a disordered to an ordered state. A similar report showed that the content of the α-helix of mung bean protein isolates increased and its β-sheets decreased during the MR with glucose [43]. In summary, Gal and βG exhibited different effects on the SPI secondary structure, which in turn can lead to functional differences.

#### 3.2.2. The Degree of Glycosylation (DG)

The DG represents the level of amino group depletion in proteins or peptides and can be used to evaluate the degree of the MR. It is also one of the most significant characteristics of MR conjugates [44]. The DG of the SPI-Gal was 18.84%, while the DG of the SPI-βG was 10.61% (Table 1). The phenomenon suggested that D-galactose can bind more amino groups of SPI, leading to a higher degree of glycosylation. This could be attributed to the monosaccharide-involved Maillard reaction system having a faster reaction rate (Section 3.1). The difference in the DG of the SPI conjugates can further affect its functional properties [45].

#### 3.2.3. Fluorescence Spectral Analysis

**Figure 3:**
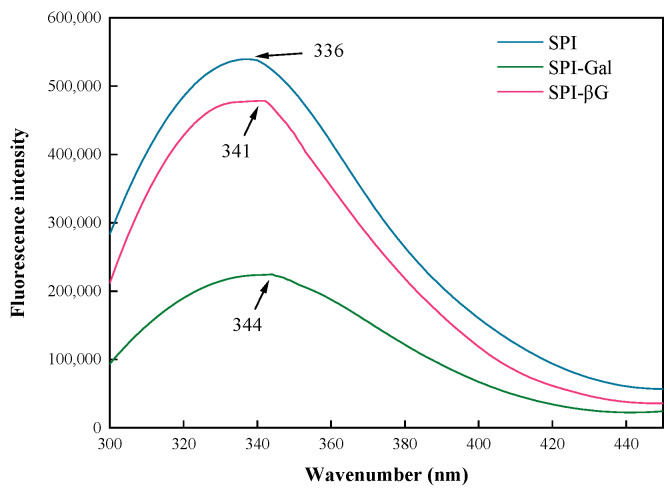
Fluorescence spectra of SPI conjugates.

Fluorescence spectroscopy is utilized to assess the conformational changes surrounding the tryptophan residues of the protein. Figure 3 showed that the MR caused a reduction in the SPI’s fluorescence intensity and a redshift in the maximum absorption peak (λ_max_) of SPI. The λ_max_ for SPI, SPI-Gal, and SPI-βG was 336, 344, and 341 nm, respectively, indicating a more pronounced redshift for the SPI-Gal, and its fluorescence absorption intensity decreases more rapidly. Changes in the fluorescence intensity and redshift in λ_max_ are sensitive indicators of protein structure changes, providing important information about protein modification [46]. The redshift in the maximum absorption peak of SPI after MR is typically associated with the exposure of more hydrophobic groups as the MR unfolds the SPI chains. This process places tryptophan and tyrosine residues in a more hydrophobic environment [24,47]. Furthermore, there is a pronounced decrease in fluorescence absorption intensity and a more significant redshift for SPI-Gal. This could be attributed to its higher DG.

### 3.3. Physicochemical Property

#### 3.3.1. Surface Hydrophobicity (*H*_0_)

The *H*_0_ is an indicator of the amount of hydrophobic groups on the protein molecular surface upon contact with a polar aqueous environment [48]. Research indicated that the majority of a protein’s hydrophobic groups are sequestered within the core of a compact, globular region [49], thereby restricting their interaction with ANS and leading to a notably lower *H*_0_ value for SPI (Table 1). The MR enhanced the *H*_0_ of SPI, potentially due to the conjugation with Gal/βG, which induced the dissociation of the SPI aggregates, resulting in the exposure of hydrophobic groups [50]. Specifically, SPI-Gal exhibited higher *H*_0_ values, indicating a stronger surface hydrophobicity. This was due to the higher DG value of the conjugates, thus exposing more hydrophobic groups.

#### 3.3.2. Solubility

**Figure 4:**
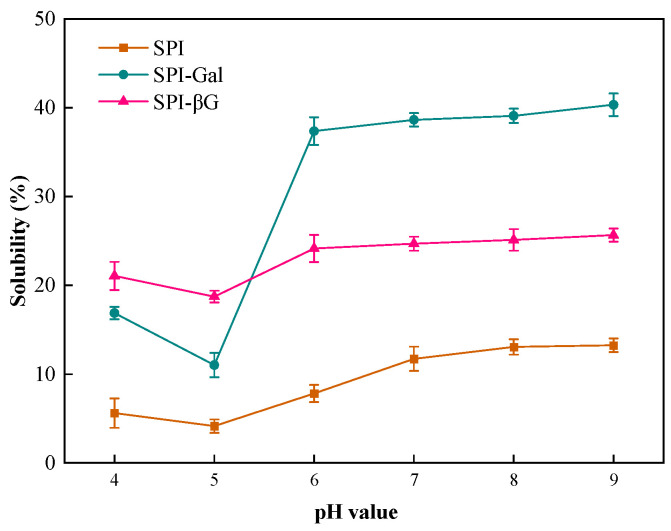
Solubility of SPI conjugates.

Solubility is an important indicator of SPI in food applications, and most functions of proteins are related to their solubility [51]. The solubility of the samples at different pH values is shown in Figure 4. Compared to SPI, the solubility of both SPI-Gal and SPI-βG was enhanced after the MR. This is because during the MR, the hydrophilic groups on the saccharide molecules that covalently bonded to SPI increased the hydrophilicity of the SPI, thereby improving its solubility in aqueous solutions [14]. Both SPI-Gal and SPI-βG showed a decrease in solubility at pH 4–5. This may be because the net charge is almost zero near the isoelectric point of SPI (pH about 4.5), which weakens the repulsive forces between molecules, leading to an easier aggregation of proteins and thus, a reduction in solubility [52]. The solubility of both SPI-Gal and SPI-βG was significantly higher compared to SPI over the entire pH range, especially at pH 6–9. Additionally, Gal had a greater ability to increase the solubility of SPI compared to βG, except near the isoelectric point of SPI. This indicated that SPI-βG has a higher solubility than SPI-Gal near the isoelectric point of SPI. In summary, the MR can improve the solubility of SPI, thereby broadening the application of SPI in the food industry.

#### 3.3.3. Emulsifying Activity (EA) and Emulsifying Stability (ES)

EA refers to the protein’s ability to form emulsion under external force, while ES is the efficacy of emulsion droplets to sustain dispersion, preventing buoyancy and aggregation within a certain time frame [53]. The EA and ES of SPI were increased by the MR (Table 2). Similar results have been reported in previous studies. For example, both SPI–citrus pectin conjugates and SPI–apple pectin conjugates increased the EA and ES of SPI as reported by Ma et al. [54]. Additionally, the conjugates of SPI with galacto-oligosaccharides also exhibited an increase in the EA and ES of SPI [55]. Compared with SPI, the SPI-Gal and SPI-βG conjugates exhibited enhanced EA in our results. This is because the hydrophilic groups of the saccharides connected to the protein increased the solubility of SPI, thereby improving and contributing to the formation of a more uniform emulsion [50]. Furthermore, the hydrophilic saccharides were linked to proteins, combining the significant adsorption of the protein at the oil–water interface with the solvation of the saccharides in the aqueous medium, resulting in an increased interfacial activity [56]. In addition, the increased ES values of the SPI conjugates may be due to the covalent binding of the saccharides to the protein, which led to the formation of a viscoelastic layer and an enhancement in the steric stabilization, resulting in the prevention of creaming, flocculation, and coalescence [56]. It can also be observed that SPI-Gal demonstrated a superior ES. This phenomenon could be due to the higher solubility and viscosity of SPI-Gal (see Section 3.3.2 and Section 3.4), which could contribute to its ES. On the other hand, SPI-βG showed better EA and the structural reasons behind this outcome required further in-depth investigation.

#### 3.3.4. Foaming Capacity (FC) and Foam Stability (FS)

**Figure 5:**
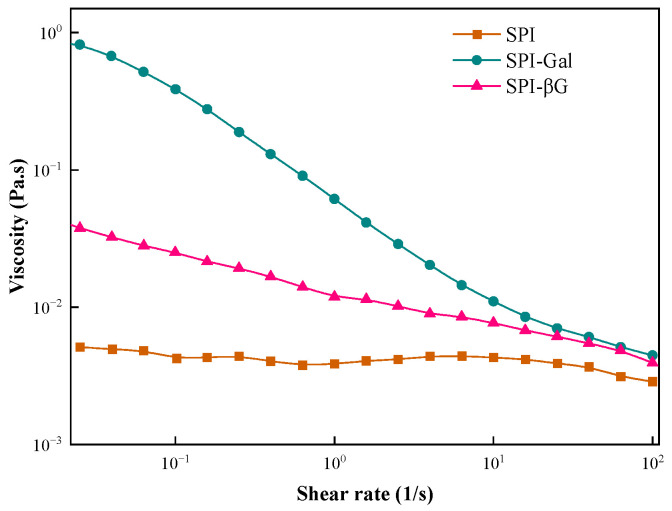
The apparent viscosity of SPI conjugates.

Proteins can adsorb rapidly to the air–water interface in stirring or bubbling, and protein molecules can quickly change their conformation and rearrange at the interface to exhibit foaming properties [57]. FS is characterized by a thick, elastic, viscous, continuous, and gas-permeable protein film surrounding each bubble [36]. In Table 2, the FC and FS of SPI were strengthened by the MR. This is due to the conjugated saccharide introducing polar groups, which strengthen the electrostatic interactions between SPI, thereby increasing the strength of the protein film [58]. Secondly, during the MR, the helical structure of SPI was unfolded, and the increased hydrophobicity (*H*_0_) can help protein adsorption at the air–water interface, while the increased flexibility enhanced its foaming properties [57]. Meanwhile, the increase in the viscosity of SPI after the MR can help stabilize the foam structure and prevent foam rupture. Compared to SPI-βG, SPI-Gal demonstrated superior FC and FS, which may be related to its higher viscosity (Figure 5) and hydrophobicity (*H*_0_) (Table 1).

### 3.4. Viscosity

The protein’s rheological characteristics are relevant to its functionality and can directly affect the quality of the food. The viscosity of SPI and its conjugates varied with the shear rate as shown in Figure 5. Within the shear rate range of 0.1–100 s^−1^, SPI is almost preferably a Newtonian fluid, while SPI-Gal and SPI-βG exhibited typical pseudoplastic fluid. This behavior was attributed to the random coil polymer that fractured and became misaligned in shearing [45]. Compared to SPI, the viscosity of the conjugates increased. This is because the covalent binding of SPI with the saccharide resulted in more branched SPI molecules, which were more prone to entanglement. Among them, SPI-Gal exhibited a higher viscosity, which may be related to the greater amount of galactose conjugated to SPI.

### 3.5. Physiological Activity

**Figure 6:**
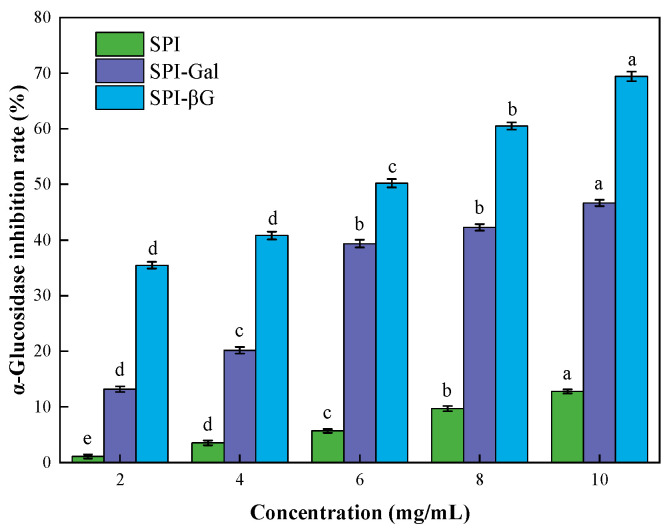
α-Glucosidase inhibitory activity of SPI conjugates. Different letters indicate statistically significant differences (*p* < 0.05).

Research indicated that soluble dietary fiber in soy can alleviate many chronic diseases, such as cardiovascular diseases and diabetes [59]. However, research on the physiological activity of SPI is still relatively limited. Therefore, the SP, FB, BAB, GA, and α-glucosidase inhibitory activity of SPI conjugates were evaluated (Table 3 and Figure 6).

SP is associated with certain bioactivities, like the hypocholesterolemic effect of dietary fiber [60]. The SP of SPI showed 9.12 g/g, which declined rapidly by MR. This might be due to the introduction of hydrophilic groups in SPI by the conjugated saccharide, enhancing the affinity between the SPI and water molecules, thus enhancing its solubility. As a result, the water retention capacity of SPI is reduced, leading to decreased SP.

FB is an essential predictor of hypolipidemic efficacy [61]. As shown in Table 3, the MR significantly enhanced the FB of SPI. During the MR, SPI was conjugated with the saccharide molecules, resulting in a greater number of hydroxyl groups on SPI. Studies indicated that hydroxyl groups perform a significant function for FB in macromolecules [62]. This might be the structural origin of the increased FB of the SPI conjugates. Compared to SPI-βG, SPI-Gal exhibited a higher FB, resulting from more hydroxyl groups on SPI-Gal. However, the underlying mechanisms needed further investigation.

BAB is an important physiological activity of SPI. Soy saponins, hydrophobic peptides, and soluble dietary fiber in SPI possess a binding capacity to bile acids, leading to the reduction in cholesterol to avoid chronic diseases [63]. The BAB of the SPI conjugates was obviously greater than that of SPI, suggesting that the MR increased the BAB of SPI. Bile acids are amphoteric substances with both hydrophilic and hydrophobic groups. During MR, the saccharide molecules introduced hydrophilic groups to SPI. As a result, the conjugates could adsorb more bile acids. Studies have shown that conjugates obtained from the MR of L-lysine with α-D-glucose can decrease plasma cholesterol by attracting bile acids [64]. Although SPI-Gal had a higher degree of glycosylation (DG) with more hydroxyl groups, SPI-βG exhibited a stronger bile acid-binding capacity, which might be associated with the inherent bile acid-binding capacity of βG itself [65].

During in vivo digestion studies, the levels of glucose released in the systems processed with SPI, SPI-Gal, and SPI-βG were measured at 20.33, 18.90, and 10.05 mmol/L, respectively. This suggested that the GA of SPI was reduced after the MR, thereby enhancing its hypoglycemic activity. Generally speaking, reduced pancreatic amylase activity and enhanced intestinal viscosity are associated with a reduction in starch digestibility and glucose absorption [66]. Although SPI-Gal showed higher viscosity, SPI-βG exhibited greater hypoglycemic activity, which may be related to the inherent good hypoglycemic activity of βG itself [67].

α-Glucosidase is a pivotal enzyme in type 2 diabetes mellitus. It is produced by the small intestine, which has the capacity to elevate blood glucose levels by transforming maltose or sucrose into glucose [68]. Carbohydrate absorption could be reduced by inhibiting α-glucosidase, consequently leading to lower blood glucose levels and preventing hyperglycemia [69,70]. The α-glucosidase inhibitory rate of SPI was enhanced by the MR (Figure 6). Research indicated that the MR conjugates obtained from chitosan and glucose can enhance α-glucosidase inhibitory activity [71]. Although SPI-Gal showed higher viscosity, SPI-βG exhibited superior α-glucosidase inhibitory activity, which might be related to the inherent good hypoglycemic activity of βG itself [67]. Our results demonstrated that MR serves as a potent approach to strengthen the hypoglycemic effect of SPI.

### 3.6. Antioxidant Activity

#### 3.6.1. DPPH Scavenging Capacity

**Figure 7:**
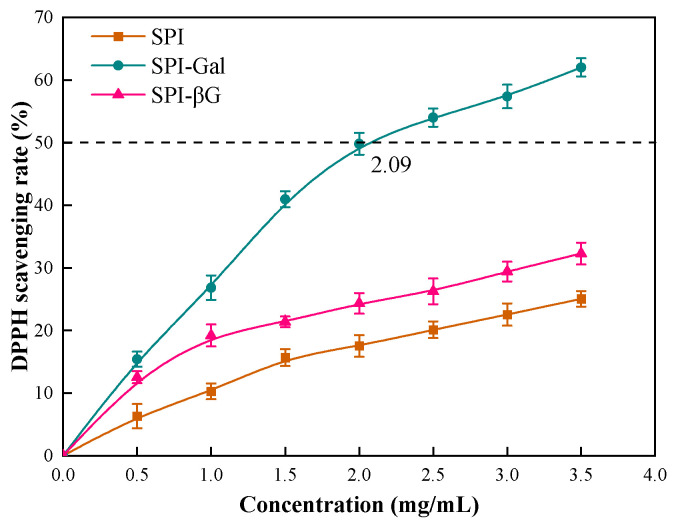
DPPH scavenging activity of SPI conjugates. The dotted line is the concentration at which the DPPH scavenging rate reaches 50%.

The DPPH scavenging capacity is a significant method to evaluate antioxidant potential [62]. Figure 7 illustrates the DPPH radical scavenging capability of SPI and its conjugates, showing an enhancement in this capability after the MR. The 50% inhibition concentration (IC_50_) of SPI-Gal against DPPH was 2.09 mg/mL. However, within the experimental range, the IC_50_ value for SPI-βG could not be determined, indicating a superior DPPH scavenging ability for SPI-Gal. Studies have shown that DPPH radical scavenging capacity correlates with the quantity and positioning of hydroxyl groups [72]. Due to the higher degree of glycosylation (DG) in SPI-Gal, Gal introduced more hydroxyl groups onto SPI compared to βG, thus endowing it with a more potent DPPH scavenging ability.

#### 3.6.2. Reducing Power

**Figure 8:**
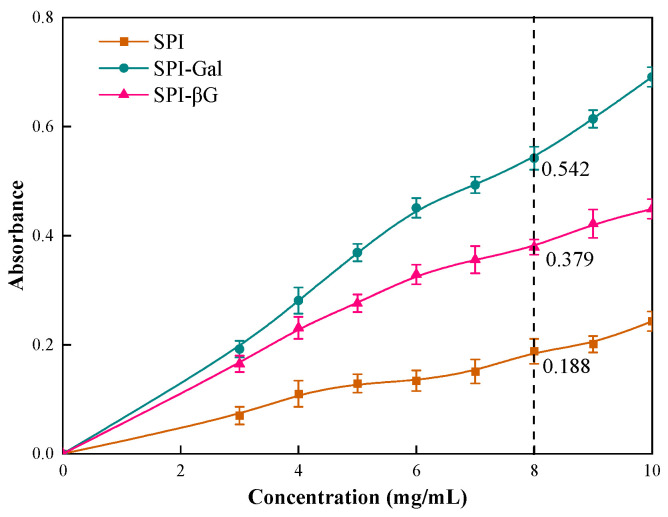
Reducing power of SPI conjugates. The dotted line is the reducing power of the samples at a concentration of 8 mg/mL.

Reducing power has a certain connection with antioxidant capacity except for free radical scavenging capacity. The reducing power of the SPI conjugates was assessed by a potassium ferricyanide reduction method [73]. The higher the absorbance of the samples, the stronger their reducing power (Figure 8). The absorbance of SPI, SPI-βG, and SPI-Gal were 0.188, 0.379, and 0.542 at an 8 mg/mL concentration, respectively. This indicated that the MR enhanced the reducing power of SPI, and SPI-Gal demonstrated superior reducing capacity. Another study has shown that the reducing power of antioxidants is correlated to their antioxidant properties [19]. Because of the higher DG for SPI-Gal, and the fact that Gal introduced more hydroxyl groups to SPI, it conferred a stronger reducing capability.

#### 3.6.3. Ferrous Ion-Chelating Capacity

**Figure 9:**
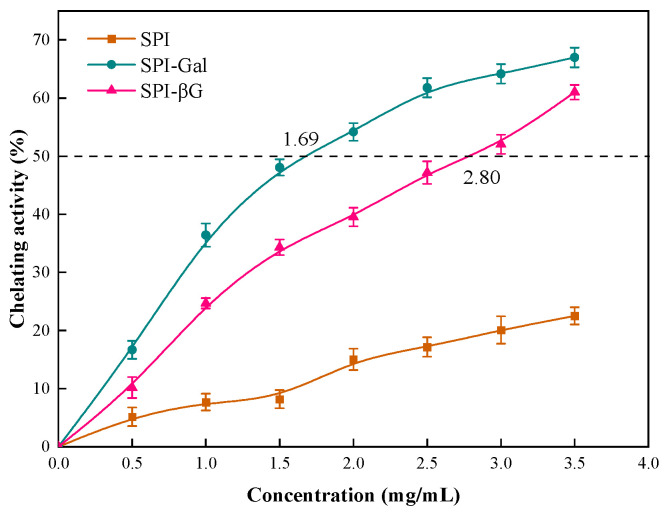
Ferrous ion-chelating capacity of SPI conjugates. The dotted line is the concentration at which the ferrous ion-chelating capacity reaches 50%.

The ferrous ion-chelating capacity reflects the capacity of antioxidants to chelate prooxidant metal ions like iron and copper, which can act as catalysts to promote oxidation [62]. As depicted in Figure 9, the concentration of SPI-Gal and SPI-βG required to achieve 50% chelation of ferrous ion (EC_50_) was 1.69 and 2.80 mg/mL, respectively, while the EC_50_ for SPI could not be determined within the experimental range. The results suggested that the MR enhanced the ferrous ion-chelating ability of SPI. Compared to SPI-βG, SPI-Gal exhibited superior ferrous ion chelation ability, which may be attributed to its higher DG and more hydroxyl groups on the molecules. Research has indicated that substances rich in hydroxyl groups possess excellent metal-chelating abilities [74]. Similar to the DPPH scavenging capacity and reducing power results, SPI-Gal exhibited a stronger ability to chelate ferrous ions.

## 4. Conclusions

During the Maillard reaction, the condensation reaction with the saccharide molecules altered the secondary structure of SPI. The introduction of the hydrophilic hydroxyl groups unfolded the compact structure of SPI, exposing a greater number of hydrophobic groups. These structural modifications resulted in a series of functional enhancements of SPI. Additionally, the type of saccharide had different effects on the structure and functionality of SPI. The reaction rate between SPI and Gal proceeded more rapidly compared to βG, resulting in a greater degree of glycosylation of SPI-Gal. This also resulted in a greater number of hydroxyl groups on SPI-Gal. Consequently, SPI-Gal exhibited superior solubility, surface hydrophobicity, and viscosity. The enhanced characteristics result in SPI-Gal possessing superior emulsifying stability, foaming capacity, and foam stability. In comparison with SPI-Gal, SPI-βG, despite its lower viscosity, showed stronger bile acid-binding capacity and hypoglycemic activity, potentially due to the inherent activity of βG. SPI-Gal exhibited superior antioxidant properties due to its higher content of hydroxyl groups on its molecules. This work will provide some scientific basis for SPI research and its further application in the functional food field.

## Figures and Tables

**Table 1:** Degree of glycosylation and surface hydrophobicity of SPI conjugates.

Sample	DG (%)	*H*_0_ Values
SPI	-	246.22 ± 3.90 ^c^
SPI-Gal	18.84 ± 0.38 ^a^	986.60 ± 7.01 ^a^
SPI-βG	10.61 ± 0.16 ^b^	600.41 ± 3.96 ^b^

Data are mean ± standard deviation. Different letters represent significant differences (*p* < 0.05).

**Table 2:** Emulsifying activity, emulsifying stability, foaming capacity, and foam stability of SPI conjugates.

Sample	Emulsifying Activity (m^2^/g)	Emulsifying Stability (min)	Foaming Capacity (%)	Foam Stability (%)
SPI	21.17 ± 0.37 ^c^	11.17 ± 0.26 ^c^	15.01 ± 0.27 ^c^	11.67 ± 0.29 ^c^
SPI-Gal	47.92 ± 0.42 ^b^	19.18 ± 0.28 ^a^	62.50 ± 0.34 ^a^	52.09 ± 0.43 ^a^
SPI-βG	57.69 ± 0.35 ^a^	13.26 ± 0.41 ^b^	37.07 ± 0.32 ^b^	38.78 ± 0.57 ^b^

Data are mean ± standard deviation. Different letters represent significant differences (*p* < 0.05).

**Table 3:** Physiological activity of SPI conjugates.

Sample	Swelling Power (g/g)	Fat-Binding Capacity (g/g)	Bile Acid-Binding Capacity (%)	Glucose Availability (mmol/L)
SPI	9.12 ± 0.19 ^a^	3.89 ± 0.13 ^c^	10.89 ± 0.28 ^c^	20.33 ± 0.27 ^a^
SPI-Gal	2.05 ± 0.07 ^b^	10.67 ± 0.18 ^a^	20.78 ± 0.32 ^b^	18.90 ± 0.14 ^b^
SPI-βG	1.10 ± 0.03 ^c^	7.50 ± 0.15 ^b^	48.96 ± 1.43 ^a^	10.05 ± 0.12 ^c^

Data are mean ± standard deviation. Different letters represent significant differences (*p* < 0.05).

## Data Availability

The original contributions presented in the study are included in the article, further inquiries can be directed to the corresponding author.
